# Low uptake of cervical cancer screening among HIV positive women in Gondar University referral hospital, Northwest Ethiopia: cross-sectional study design

**DOI:** 10.1186/s12905-018-0579-z

**Published:** 2018-06-07

**Authors:** Abebe Dires Nega, Mulat Adefris Woldetsadik, Abebaw Addis Gelagay

**Affiliations:** 1 Gondar University Hospital, Gondar City, Ethiopia; 2 College of Medicine and Health Science, Gynecology and Obstetrics Department, University of Gondar, Gondar City, Ethiopia; 3 College of Medicine and Health Science, Reproductive Health Department, University of Gondar, Gondar City, Ethiopia

**Keywords:** Cervical cancer, Screening, HIV positive women, Ethiopia

## Abstract

**Background:**

Cervical cancer is one of the leading causes of death in women worldwide. Majority of the cases are found in developing countries. The increasing risk of cervical cancer death and the high prevalence of human papilloma virus (HPV) infection in Human immuno-deficiency virus(HIV) positive women calls for determining the level of premalignant cervical cancer (Ca) screening uptake. So, this study aimed to assess the uptake of cervical cancer screening and its associated factors.

**Methods:**

An institution based cross sectional study was conducted from April to May, 2016, among adult HIV positive women attending care and treatment at Gondar University Referral Hospital. The data were collected using an interviewer administered questionnaire.

Bivariate and multivariable logistic regression analyses were used to determine the presence and the degree of association between dependent and independent variables. In the multivariable logistic analysis, a *P*-value of < 0.05 and odds ratio with a 95% confidence interval were considered to determine independent predictors for the uptake of cervical cancer(Ca) screening.

**Results:**

The life-time uptake of cervical cancer screening among HIV positive women was 10% (95% Confidence Interval(CI): 7.3–12.8). In multivariable the analysis, women with primary education (Adjusted Odds Ratio(AOR) = 3.92, 95%CI:1.70–8.99), secondary education (AOR = 3.84, 95%CI: 1.50–9.83), and tertiary level education (AOR = 4.16, 95%CI: 1.24–13.98), having a child (AOR = 3.02, 95%CI: 1.23–7.46), diagnosed as HIV positive ten years back or more (AOR = 2.71, 95% CI: 1.06–6.97), and Cell Differentiation 4(CD4) count of less than or equal to 200cell/mm3 (AOR = 5.29, 95% CI: 2.58–10.83) were significantly associated with the uptake of cervical cancer screening.

**Conclusion:**

In this study, the uptake of cervical cancer screening was very low. Educational status, parity, length of time after diagnosis as HIV positive, and CD4 count are important predictors of cervical cancer screening. Health care workers and cervical cancer prevention and control program coordinators and implementers need to provide counseling services for all Anti-retroviral Therapy(ART) care attendants. So as to explore the root causes for the low utilization of precancerous stage of cervical Ca screening service, conducting a study on the supply side with a qualitative component is mandatory.

**Electronic supplementary material:**

The online version of this article (10.1186/s12905-018-0579-z) contains supplementary material, which is available to authorized users.

## Background

Cancer is an abnormal growth of body cells [1]. Globally, cervical cancer is one of the most common (ranks fourth) cancer in women. The 2012 estimate noted that around half a million (528,000) new cervical cancer cases and more than a quarter of a million (266,000) deaths occurred due to cervical cancer globally. The burden of the disease and death from cervical cancer is very high in developing countries [2]. Cervical cancer is the second most common cancer in women living in less developed regions, with an estimated 445, 000 new cases in 2012 [3].

Globally, more than 2.7million women are at risk of cervical cancer, out of which about 80% were found in less developed regions [4]. The estimated incidence rate of cervical cancer in 2012 ranged from 4.4 per 100,000 in Western Asia to 42.7 per 100,000 in Eastern Africa [2]. In Ethiopia, cervical cancer is the second leading cause of female cancer and female cancer death. The 2012 estimate showed that the age-standardized incidence rates of cervical cancer of Ethiopia = 26.4/100,000 women. The same estimate noted that about 4732 cervical cancer deaths occurred annually and age-standardized mortality rate of the problem was 18.4/100,000 in the country [5].

Cervical cancer is caused by a sexually acquired infection with certain types of HPV. Nearly 70% of the cervical cancer (Ca) is caused by HPV(Human Papilloma Virus) of type 16 and type 18 [3, 6].

According to the 2015 report from Information Centre on HPV and Cancer, around 29.43million women (> = 15 years of age) were at risk of Cervical Ca in Ethiopia [7]. Early first sexual intercourse, multiple sexual partners, and immune suppression are the main risk factors for HPV persistence and development of cervical cancer [3]. HIV positive women have a higher incidence of persistent HPV infection which leads to an increased risk of developing premalignant lesion of the cervix. The risk of developing invasive Cervical Ca in HIV positive women is ten years earlier than HIV negative women [8].

The detection rate of pre cervical lesion was higher among HIV sero-positive women than among non-HIV infected women. Studies in USA [9], Kenya [10], and Zambia [11], reported the contrast as 5% Vs 2, 25% Vs 19, and 41% Vs 20%, respectively.

Cervical cancer is the only gynecologic cancer for which screening test is available that can detect its pre-cancerous stage. The World Health Organization recommended that cervical cancer screening is needed for women aged 30–49 years irrespective of their HIV sero-status. It is also recommended that sexually active girls and women need to be screened as soon as they are diagnosed as positive for HIV [12].

In Ethiopia, visual inspection with acetic acid(VIA) as a screening tool and cryotherapy as a treatment modality for premalignant cervical lesion are available in some hospitals [13]. The service is mainly for HIV positive women. However, there is limited information about the level of cervical cancer screening service utilization in Ethiopia in general and in the study area in particular. Therefore, this study aimed to assess the uptake of cervical Ca screening and associated factors among HIV positive women. Thus, it is expected to help policy/decision makers, non-government organizations (NGOs), health care providers, and community service providers design strategies and take necessary interventions accordingly.

## Methods

### Study design and period

An institution- based cross sectional study was conducted among HIV positive women attending adult ART (Anti-Retroviral Therapy) Clinic at Gondar University Referral Hospital, northwest Ethiopia, from April 16 to May 15, 2016.

### Study area

The study was conducted at Gondar University Referral Hospital, in the chronic HIV care and treatment clinic, Gondar. Gondar city is found in the Amhara Regional State, 727 km from Addis Ababa, the capital of Ethiopia. According to the 2007 census conducted by the Central Statistical Agency of Ethiopia, Gondar had a total population of 206, 987 and more than half (108,902) were females. The city had one teaching referral hospital, namely the University of Gondar Referral Hospital which was providing services to more than five million people. The HIV care service of the hospital was established in 2003 Gregorian calendar (GC) and has three clinics: Adult ART, pediatric ART, and Volunteer Counseling and Testing (VCT). There were about 9565 HIV positive clients enrolled in the adult ART Clinic. From these, 4731 clients were on Highly Active Antiretroviral Therapy (HAART). Before the emergence of visual inspection with acetic acid (VIA) for cervical Ca screening, pap smear was used. However, it was not routinely used for all HIV positive women. Instead, the physician ordered the pap smear only for women who were suspected for or have symptoms of cervical Ca irrespective of their HIV sero-status. Premalignant cervical cancer screening by visual inspection with acetic acid (VIA) and cryotherapy services have been available in the Hospital since 2013. In principle, the screening service for precancerous cervical lesion using VIA has to be given routinely to all HIV positive women free of charge and the screening service can be repeated every three year. However, what is actually being done is that providers selectively order/direct some women to screen. Trained nurses and physicians, who are assigned in the ART clinic, have the responsibility to provide information, education, and counseling (IEC) and the screening service.

### Study population

All HIV positive women aged 18 years and above and were receiving care in the Adult ART Clinic during the study period were the study population.

### Sample size determination

A single population proportion formula [n = (Z_α/2_)^2^ * p(1-p)/d^2^] was used to estimate the sample size. By considering the findings of a study conducted in Ethiopia and the following assumptions: proportion of cervical Ca screening uptake among HIV positive women = 12% [14], 3% margin of error, and a 95% confidence level, the required sample size was 451. Sample size was also calculated from more frequently observed associated factors for the uptake of Cervical Ca screening, like age and educational status [8, 12] using epi info StatCalc and became 52 and 192, respectively. Finally, the larger sample size of 496 was selected by considering a10% non-response rate.

### Sampling procedure

Out of all clients attending the Adult ART Chronic Follow-up Clinic, the average monthly number of women was estimated by considering the number of service users in the past six months. Using a systematic random sampling technique, every 5th woman on the list of their order of arrival for follow up care was selected and formed the participants of the study.

### Operational definitions:

- **Uptake of cervical cancer screening:** HIV positive women who were screened for premalignant cervical lesion at least once in their life time (self-reported).
- **Awareness of cervical cancer screening:** HIV positive women who had heard about cervical cancer and cervical Ca screening.

### Data collection tool and procedure

The questionnaire was prepared in English (Additional file 1) and translated to Amharic (Additional file 2). The local language, and then retranslated to English by language experts. Four first degree graduate nurses were employed for data collection. A one day training was given to data collectors on the methods, objectives, and the tool before collection began. The tool was pre-tested on 20 cases at a nearby Azezo Health Center to see the accuracy of responses, the clarity of language and the appropriateness of the tool. Every 5th woman in the order of arrival for follow up care were given information about the study and were requested to participate. After obtaining consent, data were collected using the interviewer administered questionnaire. Patients chart (medical record) was also reviewed to obtain clinical parameters, such as the WHO (World Health Organization) clinical staging for HIV infection and their CD4 count. The investigators checked the completeness of the questionnaire and conducted the overall supervision on a daily base.

### Data processing and analysis

The collected data were checked and coded manually and entered into Epi-info version 7 and then exported to Statistical Package for Social Sciences (SPSS) version 20.0 for statistical analysis. Descriptive statistics and binary logistic regression analyses were done. In the bivariate analysis, variables which had significant association with the outcome variable at 0.2 *p*-value were considered for multivariable analysis. The Back ward Wald method was employed in the multivariable analysis. In the multivariable logistic regression analysis, p-value less than 0.05 and adjusted odds ratio with a 95% confidence interval were used to determine the presence and degree of association between dependent and independent variables.

## Results

### Socio-demographic and economic characteristics of the respondents

A total of 496 HIV positive women were approached. Out of these, 460 women agreed to participate in the study (92.7% response rate). The mean age of the study participants was 35.5 years (Standard deviation (SD) = ± 8.4) and 197(42.8%) of the participants were found in the age range of 25–34 years. The majority of participants, 345(75%), were Orthodox Christians; 282(61.3%) were Amhara by ethnicity; 193(42.0%) were married; 353 (76.7%) were urban dwellers; 217(47.2%) had no formal education, and 311(67.6%) had at least one child (Table 1).

**Table 1:** Socio-demographic and economic characteristics of HIV positive women attending adult ART clinic in Gondar university referral Hospital, Northwest Ethiopia, April 2016 (*n* = 460)

Variable	Category	Frequency (%)
Age in year	18–24	28 (6.1%)
	25–34	197 (42.8%)
	35–44	164 (35.7%)
	45–54	55 (12.0%)
	>/=55	16 (3.5%)
Religion	Orthodox Christian	345 (75%)
	Muslim	74 (16.1%)
	Catholic	12 (2.6%)
	Protestant	23 (5.0%)
	Others^a^	6 (1.3%)
Ethnicity	Amhara	282 (61.3%)
	Kimant	78 (17.0%)
	Tigray	68 (14.8%)
	Oromo	13 (2.8%)
	Others^b^	19 (4.1%)
Residence	Urban	353 (76.7%)
	Rural	107 (23.3%)
Educational status	No formal education	217 (47.1%)
	Primary	126 (27.4%)
	Secondary	85 (18.5%)
	Tertiary	32 (7.0%)
Marital status	Single	120 (26.0%)
	Married	193 (42.0%)
	Divorced	62 (13.5%)
	Widowed	85 (18.5%)
Number of child /parity/	Having child	311(67.6%)
	Don’t have a child	149 (32.4%)
Occupation	Self employed	154 (33.5%)
	House wife	102 (22.2%)
	Not employed	74 (16.1%)
	Government employee	40 (8.7%)
	Daily laborer	41 (8.9%)
	Employee in private organization	36 (7.8%)
	Others^c^	13 (2.8%)
Monthly income (in USD)	< 35	119 (25.8%)
	35–50	110 (23.9%)
	51–100	113 (24.6%)
	> 100	118 (25.7%)

Others^a^ = Adventist and Jewish; Others^b^ = SNNP and Gambella; Others^c^ = student and retired

### Awareness of the study participants and their clinical profile

Half of the participants, 228 (49.6%), heard about cervical cancer, and 174 (37.8%) heard about premalignant cervical cancer screening. The majority, 409 (88.9%), of the respondents were on a highly active antiretroviral therapy (HAART), and 388 (84.3%) had CD4 count of > 200/mm^3^. Slightly more than a half(53.9%)and nearly one third (32%) of the participants were in WHO clinical stage I and II respectively. Very few (3%) respondents were in stage IV.

### Uptake of cervical cancer screening

In this study, the life time uptake of premalignant cervical cancer screening service was 10% (95% CI: 7.3–12.8), of which 43 (93.4%) were screened after diagnosis for HIV. Among those screened, 40(86.9%) were screened in the past three years and 7(15.2%) stated that premalignant cervical lesion was detected in their cervix. Among those who were screened, all the 46 (100%) used the screening service up on health service providers’ request.

### Factors associated with the uptake of cervical cancer screening

Independent variables which have been considered for a bivariate analysis were socio-demographic variables (age, religion, ethnicity, residence, educational status, marital status, number of child /parity/, occupation, monthly income), client’s awareness, ART status, duration (in year) after diagnose of HIV, time since enrolled to HIV care (in year), and CD4 count. Among these variables, number of child /parity/, age, residence, educational status, marital status, monthly income, duration (in year) after diagnose of HIV, time since enrolled to HIV care (year), and CD4 count had a *p*-value of less than 0.2 in the bivariate analysis. Thus, these variables were considered for the multivariable analysis. In the multivariable analysis, HIV positive women who had children, those who attended formal education, women whose HIV status was diagnosed ten years ago, and women whose CD4 cell count was less than or equal to 200cell/mm3 had statistically significant association with uptake of Cervical Ca screening (Table 2).

**Table 2:** Bivariate and multivariable analysis for the uptake of Cervical Ca screening among HIV positive women attending adult ART clinic in Gondar university referral hospital, Northwest Ethiopia, April 2016 (*n* = 460)

Variables	Category	Cervical Ca screening uptake		COR(95%CI)	AOR(95%CI)
		Yes	No		
Parity	0	7	142	1.00	1.00
	>/=1	39	272	2.90(1.267–6.67)^a^	3.02(1.22–7.46)^a^
Residence	Rural	5	102	1.00	
	Urban	41	312	2.68(1.03–6.97)	
Age in year	18–24	1	28	0.08(0.01–1.05)	
	25–34	10	186	0.12(0.04–0.44)^a^	
	35–44	24	140	0.38(0.12–1.18)	
	45–54	6	49	0.27(0.07–1.04)	
	>/=55	5	11	1.00	
Educational Level	No formal education	12	205	1.00	1.00
	Primary	18	108	2.85(1.32–6.13)^a^	3.92(1.70–8.99)^a^
	Secondary	11	74	2.54(1.07–6.00)^a^	3.84(1.50–9.83)^a^
	Tertiary	5	27	3.16(1.03–9.67)^a^	4.16(1.24–13.98)^a^
Marital status	Single	6	114	1.00	
	Married	22	171	2.44(0.96–6.22)	
	Divorced	8	54	2.82(0.93–8.52)	
	widowed	10	75	2.53(0.88–7.26)	
Monthly income (in USD)	< 35	9	110	1.00	
	35–50	7	103	0.83(0.29–2.31)	
	51–100	15	98	1.871(0.78–4.47)	
	> 100	15	103	1.78(0.75–4.24)	
Diagnosed for HIV(year)	< 5	23	250	1.00	1.00
	5–9	13	134	1.06(0.52–2.15)	0.68(0.32–1.47)
	> = 10	10	30	3.62(1.58–8.34)^a^	2.72(1.06–6.97)^a^
Since enrolled to HIV care (year)	1–4	22	253	1.00	
	5–9	17	140	1.39(0.72–2.72)	
	> = 10	7	21	3.83(1.47–10.01)^a^	
CD4 count (cell/mm^3^)	<=200	19	53	4.79(2.49–9.22)^b^	5.29(2.59–10.83)^b^
	> 200	27	361	1.00	1.00

^a^*P*-value: 0.05–0.001; ^b^ P-value: < 0.001

The study participants who had children were three times (AOR = 3.02, 95%CI:1.22–7.46) more likely to be screened for cervical Ca than those who had no children. The odds of being screened for Cervical Ca among HIV positive women were four times (AOR = 3.92, 95%CI:1.70–8.99) higher in women on primary level education, four times (AOR = 3.84, 95%CI: 1.50–9.83) higher in women on secondary level education, and four times (AOR = 4.16, 95%CI: 1.24–13.98) higher in women on tertiary level education as compared to those who had no formal education.

HIV positive women whose HIV diagnosis was made ten years or more back were nearly three times (AOR = 2.71, 95% CI:1.06–6.97) more likely to be screened for cervical Ca than those whose HIV diagnosis was made within the past five years (< 5 years). Women who had less than or equal to 200cell/mm3 CD4 count were five times (AOR = 5.29, 95% CI:2.58–10.83) more likely to be screened for cervical Ca than those who had more than 200cell/mm3 CD4 count (Table 2).

## Discussion

Cervical screening for precancerous lesion is recommended especially for high risk people, like HIV positive individuals so as to take appropriate measures timely. In this study, the uptake of premalignant cervical cancer screening among HIV positive women was 10% which is nearly similar to that of the studies conducted in Addis Ababa 12% [14] and Nigeria 9% [15]. However, this finding is lower than the study findings in Kenya 84% [9], South Africa 32% [10], Italy 91% [11] and 54% [16], Canada 82% [17], and USA 84% [18], 78% [19], 85.6% [20]. This variation could be due to different socio demographic and economic status of the study participants as well as the countries. For example, the educational level of the respondents and their level of awareness about cervical Ca and its screening program were very low in this study. Though not studied, level of health service providers’ commitment in providing information, education, and counseling (IEC) and the screening service might be the reason for low uptake the screening service. Another possible reason for the difference between this study and South Africa’s study might be the different data collection technique used. In contrast to ours, self-administered questionnaire was used in South Africa. In Kenya, the availability of free cervical cancer screening program, prevention services and continuous provision of health education about cervical cancer and screening in the area could be the main reasons for the high uptake of Cervical Ca screening. The difference might also be the length of time since the establishment of Cervical Ca screening service and the integration of premalignant cervical cancer screening with HIV care in high resource countries. In Ethiopia, cervical cancer screening sites were very few and limited to comprehensive specialized hospitals, and cervical Ca screening service was initiated just two years back, and continuous and organized health education and awareness creation programs were not well established.

In this study, factors significantly associated with the uptake of cervical cancer screening among HIV positive women were educational status, parity, length of time since HIV was diagnosed, and recent CD4 cell count (Table 2). Educated women can have a better awareness about cervical cancer and its screening, mostly have decision making power, and better health care seeking initiative than non-educated which in turn can result in the utilization of the screening service. In this study, HIV positive women who had formal education (primary, secondary and tertiary) were nearly four times more likely to be screened for Cervical Ca as compared to women who did not attend formal school. The same association was observed in a study done in Italy [11].

HIV positive women who had children were three times more likely to be screened for cervical cancer than those who had no children. This could be due to the fact that women who had children might visit health care facilities more frequently in addition to their routine HIV care follow up than nulliparous. As a result, they might have chances of getting information and advice to use the screening service from health professionals.

HIV positive women with recent CD4 count of </=200 cell/mm3 were five times more likely to be screened for cervical Ca than those whose CD4 cell count was > 200 cell/mm3. Women with lower CD4 count might have advanced WHO clinical stage, they might have been diagnosed as HIV positive a long time ago and might have frequently developed opportunistic infections. Thus, they might make frequent health institution visits and be advised or counseled to be screened for cervical Ca. However, this finding contradicts the finding of a study conducted in Emilia-Romagna, Northern Italy [11].

Women who were diagnosed as HIV positive ten years back or more were nearly three times more likely to be screened for cervical Ca than women who were diagnosed in the past five years. Such women might attend health care for longer duration and might have repeated contact with health care providers for the routine HIV care and treatment services as well as for other medical conditions. Hence, they could have the chance of getting information from health care providers and peers and have better awareness about cervical cancer and its screening, which might in turn make them have better utilization of the screening service. This is in line with a study done in Kenya [9] and Northern Italy [11].

The possible limitation of this study could be the non generalizability of the findings to all HIV positive women attending care and treatment for HIV infection in public health institutions in Gondar city since the data were taken from clients attending only the Gondar University Referral Hospital where a cervical cancer screening program is available. Additionally, the authors did not assess factors from the heath service providers’ side, for example, whether they provided information and invited all HIV positive women for screening or not.

## Conclusions

In this study, the uptake of cervical cancer screening was very low. Educational status, parity, number of years after being diagnosed as HIV positive, and CD4 count were predictors of cervical cancer screening. Health care workers and cervical cancer prevention and control program coordinators and implementers need to integrate the screening service to the routine care and treatment and provide counseling services. It is also important to increase women’s awareness about the disease and the importance of screening. So as to explore the root causes for the low utilization of precancerous stage of cervical Ca screening service, conducting a study on the supply side with a qualitative component is mandatory.

## Additional files

Additional file 1: English version of the questionnaire. (DOCX 38 kb)

Additional file 2: Amharic version of the questionnaire. (DOCX 43 kb)

## Abbreviations

AOR: Adjusted Odds Ratio
ART: Anti-Retroviral Therapy
Ca: Cancer
CD4: Cell Differentiation 4
CI: Confidence Interval
HAART: Highly Active Antiretroviral Therapy
HIV: Human Immunodeficiency Virus
HPV: Human Papilloma Virus
IEC: Information, Education, and Counseling
NGOs: None Governmental Organizations
SD: Standard Deviation
SPSS: Statistical Package for Social Science
USA: United State of America
VCT: Voluntary Counseling and Testing
VIA: Visual Inspection with Acetic Acid
WHO: (World Health Organization)
