# Supplementary material for: Low uptake of cervical cancer screening among HIV positive women in Gondar University referral hospital, Northwest Ethiopia: cross-sectional study design
Source: BMC Womens Health. 2018 Jun 7;18:87. doi: 10.1186/s12905-018-0579-z (PMC5992703; doi:10.1186/s12905-018-0579-z)
Supplement: Supplementary file 1 — English version of the questionnaire. (DOCX 38 kb) [file 12905_2018_579_MOESM1_ESM.docx]

Consent form

Good morning / good after noon;

My name is___________, I am here to collect information from you about cervical cancer and related issues for the study entitled "Uptake Of Cervical Cancer Screening Among HIV Positive Women In Gondar University Referral Hospital, Northwest Ethiopia". As to the information given ahead, participating in this study has no any risk. Your name will not be written on this form and the information you give will never be shared to others. You may not answer any questions that you don't want to answer and you may end this interview at any time you want. Now I would like to tell you that you are selected randomly to be participant of the study. Your genuine response to the interviews will be very important for the purpose of the study and we would like to appreciate your voluntarily participation in the survey.

Thank you for your cooperation and listening!!!

Are you willing to participate?

Yes No (stop the interview)

##

**Questionnaire**

Code number----------------

**Part**: **I. Questions to assess socio-demographic and economic status of WLHIV.**

| S.No | Questions | Responses |
| --- | --- | --- |
| 101 | How old are you? | ____________years |
| 102 | What is your religion? | 1.Orthodox (1)  2.Muslim (2)  3.Protestant (3)  4. Catholic (4)  5.Others (5)___________ |
| 103 | What is your ethnicity? | 1.Amhara  2.Tigray  3.Oromo  4. kemant  5. Others__________ |
| 104 | Where is your place of residence? | 1.Urban  2.Rural |
| 105 | What is your marital status? | 1.Single  2.Married  3.Divorced  4.widowed |
| 106 | What is your educational status (the highest grade you completed)? | 1.unable to read and write  2.Able to read and write  3.Primery education  4.Secondary(9-12)  5.Certificate and diploma  6.colledge or university degree |
| 107 | How many children do you have? | ____________in number |
| 108 | If married in ***Q. 105 is Married,*** What is your husband’s educational status? | 1.unable to read and write  2.Able to read and/write  3.Primery education  4.Secondary(9-12)  5.Certificate and diploma  6.colledge degree and above |
| 109 | What is your occupation/ what kind of work do you mainly do/? | 1.Not employed  2. civil servant/Government employee  3. Employee in private organization  4. self employed  5. house wife  6. Student  7. Daily laborer  7.Others(specify)________ |
| 110 | What is your monthly income | ___________ (in Ethiopian birr) |

**Part II. Questions assessing HIV*/AIDS and related factors***

| S.No | Questions | Responses | Notice |
| --- | --- | --- | --- |
| 201 | How many years since you are diagnosed as HIV positive? | -------------- in year |  |
| 202 | How many years since you are enrolled in HIV care clinic? | -------------- in year |  |
| 203 | Are you on HAART or not? | 1. Yes  2. No |  |
| 204 | ***If yes to Q 203***.How many years since you are initiating HAART? | -------------- in year |  |
| 205 | Recent CD4 count |  | By document review |
| 206 | WHO clinical stage |  |  |

**Part III. Questions assessing awareness of WLHIV about premalignant CCA screening and associated factors.**

| S.No | Questions | Responses | notice |
| --- | --- | --- | --- |
| 301 | Have you ever heard about  Cervical cancer?  ***If no go to part 4*** | Yes.............1  No..............2 |  |
| 302 | From which source you have heard about cervical cancer for the first time? | Media( TV and radio)...............1  Printed materials.......................2  Health care workers...................3  Family------------------------4  friends, ……………………5  neighbors.………………….6  Other (please explain).................7 |  |
| 303 | Have you ever heard about pre malignant cervical cancer screening?  ***If no go to part 4*** | Yes.............1  No...............2 |  |
| 304 | From which source you have heard about premalignant CCa screening for the first time? | Media( TV and radio)...............1  Printed materials.......................2  Health care workers...................3  Family..............................……..4  friends.............................……..5  neighbors.................................6  Other (please explain).................7 |  |
| 305 | Do you know premalignant cervical cancer screening tests?”  ***If no go to part 4*** | Yes.............1  No...............2 |  |
| 306 | Which screening method do you know? | 1. VIA 2. Pap smear 3. Other( specify…….) |  |

|  |
| --- |

**Part IV. Questions on the uptake of pre malignant cervical cancer screening and associated factors in WLHIV.**

| S.No | Questions | Responses | Notice |
| --- | --- | --- | --- |
| 401 | Have you ever been screened for premalignant cervical cancer?  ***If yes go to Q.403*** | Yes...................1  No....................2 |  |
| 402 | If still you were not screened what was your main reason of not being screened? | I am not informed / I don’t know ...........1  I feel it is painful. ..................2  Cultural and/or religious reasons......3  I feel healthy/or no symptom..............4  I don’t know where to screen......5  it is expensive..........6  Fear of test result .........7  My husband would not agree...........8  Other /specify/………………… 9 |  |
| 403 | What was your reasons or initiates you for screening? | Self-initiated....................1  Initiated by the health professionals……2  Initiated by husband or…………3  Initiated by family……………….4  other(specify) ..................5 |  |
| 404 | What was Your Age when you have been screened for the first time? | ------------- in year of age |  |
| 405 | Where did you screened? | Public health facility….1  Private health facility…2  Other specify…3 |  |
| 406 | When did your first screening test done in relation to your sero status? | Before diagnosis of HIV………1  After diagnosis of HIV………..2 |  |
| 407 | ***If after HIV diagnosed in Q 406***.How many times you have been screened? | --------------in number |  |
| 408 | When was the last time you has been screened? | within the past three years………1  More than three years ago............2 |  |
| 409 | Do you have a reported history of abnormal screening test result? | Yes...................1  No....................2 |  |

Thank you for your cooperation

Name of data collector_______________________ signature_____________

Name of Supervisor_______________________ signature________________
