# Supplementary material for: Low uptake of cervical cancer screening among HIV positive women in Gondar University referral hospital, Northwest Ethiopia: cross-sectional study design
Source: BMC Womens Health. 2018 Jun 7;18:87. doi: 10.1186/s12905-018-0579-z (PMC5992703; doi:10.1186/s12905-018-0579-z)
Supplement: Supplementary file 2 — Amharic version of the questionnaire. (DOCX 43 kb) [file 12905_2018_579_MOESM2_ESM.docx]

## የፈቃደኝነት መጠየቂያ ቅጽ

ጤና ይስጥልኝ!

ስሜ_______________ ይባላል፡፡ ወደ እርስዎ የመጣሁት እርስወ ስለማሕጸን በር ካንሰር ቅድመ-ምርመራ ያለወትን የቅድመ ምርመራ ተሳትፎ እና ተያያዥነት ያላቸው ጉዳዮች ላይ መረጃ ለማሰባሰብ ነው፡፡

ከላይ እንደተገለጠለወት በጥናቱ ለመሳተፍ ወይም ያለመሳተፍ (እራስዎን ከጥናቱ የማግለል) መብትዎ የግል ምርጫዎ ብቻ ይሆናል፡፡ ለዚህ ጥናት ተብሎ ከእርስዎ የተገኘ ማንኛውም መረጃሚ ስጥራዊነቱ የተጠበቀ ይሆናል፡፡ በዚህ ጥናት ምክንያት ሊደርስብዎ የሚችል ምንም ዓይነት የአደጋ ተጋላጭ ወይም ጎንዮሽ ጉዳቶች አይኖሩም፡፡ እስካሁን በነበረን ቆይታ ይሳተፉበት ዘንድ ስለተጠየቁት ጥናት አስፈላጊ የተባሉትን መረጃዎች በሙሉ ገልጸንልዎታል፡፡ በዚህም መሰረት እርስወ በጥናቱ ውስጥ ተካተዋል:: መመለስ የማይፈልጉትን ጥያቄ እንዲመልሱ አይገደዱም፡፡ ለዚህ ጥናት በሚያደርጉት ትብብር ለጥንቱ መሳክት አስፈላጊ በመሆኑ በቂ መረጃ እንዳገኙ እና እንደተረዱ እያመን በፈቃደኝነት በጥናቱ ላይ ስለሚሳተፉ አመሰግናለሁ!! ስላዳመጡም አመሰግናለሁ!!

በዚህ ጥናት ለመሳተፍ ፈቃደኛ ነዎት?

አዎ ………………. ፈቃደኛ አይደለሁም ..................(መጠይቁን ያቋርጡ)

መጠይቅ

የመጠየቅ ኮድ ቁጥር____________________ ቀን………………….

**ክፍልአንድ፡** ኤች. አይ. ቪ. ቫይረስ በደማቸዉ ዉስጥ ያለባቸዉን ሴቶች የማህበራዊ፤የአኗኗር ሁኔታና የገቢ ሁኔታ የሚዳስስ መጠይቅ

| **ተ.ቁ** | **ጥያቄ** | **መልሶች ( አማራጮች)** |
| --- | --- | --- |
| 101 | እድሜዎ ስንት ነው ? | _________ /በአመት/ |
| 102 | ሃይማኖዎ ምንድን ነዉ? | 1. ኦርቶዶክስ  2. ሙስሊም  3. ካቶሊክ  4. ፕሮቴስታንት  5. ሌላ ይገለጽ ---------- |
| 103 | ብሄርዎ ምንድ ንነዉ? | 1. አማራ  2.ትግሬ  3. ቅማንት  4. ኦሮሞ  5. ሌላይገለጽ--------- |
| 104 | የሚኖሩት የት ነው? | 1. ከተማ  2.ገጠር |
| 105 | የትዳርዎ ሁኔታ ምንድን ነው? | 1. ያላገባች  2. ያገባች  3. የፈታች  4. ባለቤቷ የሞተባት |
| 106 | የትምህርት ደረጃዎ ምን ያክል ነዉ  / እስከስንት ተምረዋል? | 1.ማንበብና መጻሐፍ የማትችል 4. የሁለተኛ ደረጃ ት/ት ያላት (9-12)  2.ማንበብና መጻሐፍ የምትችል 5. ሰርተፍኬት / ዲፕሎማያላት  3. የመጀመሪያ ደረጃ ት/ት ያላት (1-8) 6.ዲግሪና ከዛ በላይ |
| 107 | ስንት ልጆች አሉዎት? | ____________በቁጥር |
| 108 | **ለተ.ቁ 105** ምላሽዎ **ያገባች** ከሆነ የባለቤትዎ የት/ት ደረጃ ምንድን ነው? ***ለሌሎቹ እለፍ*** | 1.ማንበብና መጻሐፍ የማይችል  2.ማንበብና መጻሐፍ የሚችል  3. የመጀመሪያ ደረጃ ት/ት ያለዉ (1-8)  4. የሁለተኛ ደረጃ ት/ት ያለዉ (9-12)  5. ሰርተፍኬት / ዲፕሎማ ያለዉ  6.ዲግሪና ከዛ በላይ |
| 109 | የተሰማሩበት የስራመስክ (**ዋናስራዎ**) ምንድን ነው? | 1. ስራ የሌላት  2. የመንግስት ሰራተኛ  3. የግለሰብ ድርጅት ተቀጣሪ  4. የግል ስራ ያላት  5. የቤት እመቤት  6. ተማሪ  7. የቀን ሰራተኛ  8. ሌላ ካለ ይገለጽ------- |
| 110 | የወር ገቢዎ ምን ያክል ይሆናል ? | --------------- ብር |

**ክፍል ሁለት፤ የኤችአይቪ/ ኤድስ እና ተያያዠ ጉዳዮችን የሚዳስስ መጠይቅ.**

| **ተ.ቁ** | **ጥያቄ** | **መልሶች ( አማራጮች)** | |
| --- | --- | --- | --- |
| 201 | ቫይረሱ በደምዎ ዉስጥ መኖሩ ከታወቀ ምን ያክል አመት ሆነዎት ? | …… አመት | |
| 202 | ከጤና ተቐም የኤችአይቪ/ኤድስ ክትትል ከጀሩ ምን ያክል አመት ሆነዎት ? | …… አመት | |
| 203 | አሁን የፀረ ኤች አይቪ መድሀኒት መዉሰድ ጀምረዋል? | 0. የለም 1. አዎ | |
| 204 | **ለ ተ.ቁ 203 ምላሽዎ አዎ** ከሆነ የፀረ ኤችአይቪ መድሀኒት መዉሰድ ከጀመርሽ ምን ያክል አመት ሆነሽ ? | ……… አመት | |
| 205 | የቅርብ ጊዜ የሲዲፎር (CD4) መጠን? | ………. በቁጥር | **ከካርድ ታይቶ የሚሞላ** |
| 206 | ያሚገኙበት የህመም ደረጃ በ አለም ጤና ድርጅት መስፈርት መሰረት ( WHO clinical staging) | ---------- |  |

**ክፍል ሶስት፤ የኤች አይቪ ቫይረስ በደማቸዉ ዉስጥ ያለባቸዉ ሴቶች ስለማህፀን በር ካንሰር ቅድመ ምርመራ ያላቸዉን ግንዛቤ እና ተያያዠ ጉዳዮችን መመዘኛ መጠይቅ.**

| **ተ.ቁ** | **ጥያቄ** | **መልስ** |
| --- | --- | --- |
| 301 | የማህፀን በር ካንሰር በሽታ ሲባል ሰምተዉ ያዉቃሉ?  ***አላዉቅም ከሆነ ወደ ክፍል 4 ይሂዱ.*** | 1. አዎ  2. አላዉቅም |
| 302 | ለመጀመሪያ ጊዜ ስለማህፀን በር ካንሰር የሰሙት ከምንድን ነዉ? | 1. ከመገናኛ ብዙሀን (ቴሌቪዥን፣ሬድዎ)  2. ከበራሪ ወረቀቶች፣  3. ከጤና ባለሙያወች መፅሀፍ 4. ከቤተሰብ  5. ከጎደኛ  6. ከጎረቤት  7. ሌላ ካለ ይገለፅ........... |
| 303 | ስለማህፀን በር ካንሰር **ቅድመ ምርመራ** ሰምተዉ ያዉቃሉ?  ***አላዉቅም ከሆነ ወደ ክፍል 5ይሂዱ.*** | 1. አዎ  2. አላዉቅም |
| 304 | ለመጀመሪያ ጊዜ ስለማህፀን በር ካንሰር ቅድመ ምርመራ የሰሙት ከምንድን ነዉ? | 1. መገናኛ ብዙሀን( ቴሌቪዥን፣ሬድዎ)  2. ከበራሪ ወረቀቶች፣ መፅሀፍ  3. ከጤና ባለሙያወች  4. ከቤተሰብ  5. ከጎደኛ  6. ከጎረቤት  7. ሌላ ካለ ይገለፅ........... |
| 305 | የማህፀን በር ካንሰር ቅድመ ምርመራ ዘዴ ያዉቃሉ?  ***አላዉቅም ከሆነ ወደ ክፍል 4 ይሂዱ.*** | 1. አዎ  2. አላዉቅም |
| 306 | የትኛወቹን የማህፀን በር ካንሰር ቅድመ ምርመራ ዘዴ/አይነቶች/ያዉቃሉ? | 1. ቪ. አይ. ኤ /VIA/  2. ፓፕ እስሚር( Pap smear)  3. ሌላ ካለ ይገለፅ------- |

**ክፍል አራት፤** የኤች. አይ. ቪ. ቫይረስ በደማቸዉ ዉስጥያለባቸዉሴቶችሰለማህፀንበር ካንሰር ቅድመ ምርመራ ያላቸዉን ተሞክሮ/ተሳትፎ/ እናተያያዠ ጉዳዮችን መመዘኛ ጥያቄወች.

| **ተ.ቁ** | **ጥያቄወች** | **መልስ** |
| --- | --- | --- |
| 401 | የማህፀን በር ካንሰር ቅድመ ምርመራ አድርገዉ ያዉቃሉ?  ***መልስዎ አዎ ከሆነ ወደቁጥር 403 ይሂዱ*** | 1. አዎ  2. አላዉቅም |
| 402 | *ከላይ ለተነሳዉ ጥያቄ መልስዎ* ***አላዉቅም*** *ከሆነ* እስካሁን የማህፀን በር ካንሰር ቅድመ ምርመራ ያላደረጉበት **ዋና ምክንያት** ምንድን ነዉ? | 1. ስለማላዉቅ  2. ያማልብየስለማስብ  3. በባህል / በእምነትተፅእኖ  4. የህመምስሜትስለማይሰማኝ  5. አገልግሎቱየትእንደሚሰጥስለማላዉቅ  6. ዉድነዉ ብየ ስለማስብ  7. የምርመራዉን ዉጤት ስለምፈራ  8.ባለቤቴ/ ቤተሰቦቸ ስለማይፈቅዱልኝ  9.ሌላ /ይገለፅ/............ |
| 403 | የማህፀን በር ካንሰር ቅድመ ምርመራ ለማድረግ ምን አነሳስዎ? | 1. በራሴ ተነሳሽነት  2. በጤና ባለሙያወች አነሳሽነት  3. በባለቤቴ አነሳሽነት  4. በቤተሰቦቸ አነሳሽነት  5.ሌላ /ይገለፅ/…… |
| 404 | ለመጀመሪያ ጊዜ ቅድመ ምርመራ ሲያደርጉ ስንት አመትዎ ነበር ? | …………. እድሜ በአመት |
| 405 | የማህፀን አንገት ካንሰር ቅድመ ምርመራ ያደረጉት የትነዉ? | 1. ከመንግስት የጤና ተቐም  2.ከግል የጤና ተቐም  3. ከሌላ /ይገለፅ/............ |
| 406 | ለመጀመሪያ ጊዜ ቅድመ ምርመራ ያደረጉት ኤች. አይ. ቪ. ቫይረስ በደምዎ ዉስጥ ከተገኘ በኃላ ወይ ስበፊት ነዉ? | 1. ኤች. አይ. ቪዉ ከመታወቁ በፊት  2. ኤች. አይ. ቪዉ ከታወቀ በኃላ |
| 407 | ለ ***ተ.ቁ 506 መልስዎ ኤች. አይ. ቪዉ ከታወቀ በኃላ ከሆነ*** ስንትጊዜ ቅድመ ምርመራ አድርገዋል? | -----------በቁጥር |
| 408 | ለመጨረሻ ጊዜ የማህፀን በር ካንሰር ቅድመ ምርመራ መቸ አደረጉ? | 1. ባለፈዉ ሶስት አመት  2. ከሶስት አመት በፊት |
| 409 | ካደረግሻቸዉ ቅድመ ምርመራወች ከጤና ባለሙያወች የተነገረዎት ቅድመ ካንሰርን የሚያሳይ ዉጤት/ ምልከት/ነበር ? | 1. አለ  2. የለም |

ጊዜዎትን ሰዉተዉ መረጃ ስለሰጡኝ አመሰግናለሁ

የመረጃ ሰብሳቢው ስም ………………. ፊርማ ……………

የተቆጣጣሪው ስም …………………. ፊርማ ……………….
